# Assessing surrogate heterogeneity in real world data using meta-learners

**DOI:** 10.1515/jci-2025-0033

**Published:** 2026-02-23

**Authors:** Rebecca Knowlton, Layla Parast

**Affiliations:** Department of Statistics and Data Sciences, University of Texas at Austin, Austin, USA

**Keywords:** surrogate markers, heterogeneity, observational data, meta-learners, treatment effect, 62D20, 62E20, 62P10, 62F40

## Abstract

Surrogate markers are most commonly studied within the context of randomized clinical trials. However, the need for alternative outcomes also extends to real-world public health and social science research, where randomized trials are often impractical. While standard methods for evaluating surrogate markers largely rely on the assumption of randomized treatment, there is a significant gap in applying these techniques to observational data, where the central challenge shifts to managing confounding. The few methods that do allow for non-randomized treatment/exposure do not offer a way to examine surrogate heterogeneity with respect to patient characteristics. In this paper, we propose a framework to assess surrogate heterogeneity in non-randomized data and implement this framework using meta-learners. Our approach allows us to quantify heterogeneity in surrogate strength with respect to patient characteristics while accommodating confounders through the use of flexible, off-the-shelf machine learning methods. In addition, we use our framework to identify covariate profiles where the surrogate is a valid replacement of the primary outcome. We examine the performance of our methods via a simulation study and application to examine heterogeneity in the surrogacy of hemoglobin A1c as a surrogate for fasting plasma glucose.

## 1 Introduction

The increased use of surrogate markers has been an important advancement in clinical trials, offering a pathway to more efficient and cost-effective research for complex diseases like cancer and AIDS [1], 2]. A surrogate marker is formally defined as a person-level measure that serves as a substitute for a direct measure of a primary outcome, facilitating the evaluation of treatment or exposure effects. While surrogate markers are most commonly studied within the context of randomized clinical trials, the need for alternative outcomes also extends to real-world i.e., non-randomized, observational, public health and social science research, where randomized trials are often impractical [3], 4]. Without the assumption of randomized treatment, there is a significant gap in applying surrogate evaluation methods to observational data, where the central challenge shifts to managing confounding.

### 1.1 Related work

Research on identifying surrogates in non-randomized data is scarce, as available statistical approaches for evaluating surrogate markers tend to rely on the assumption that treatment is randomized. While surrogate evaluation and mediation share similarities, and much has been done in the area of mediation with non-randomized treatments, important distinctions remain between the two approaches; we discuss this further in the Discussion. In the context of surrogate evaluation, Han et al. [5] recently proposed an approach to identify surrogate markers in real-world data by quantifying surrogate strength using the proportion of the treatment effect (PTE) on the primary outcome that is explained by the treatment effect on the surrogate with estimation using inverse probability weighting and doubly robust estimators. Agniel et al. [6] offered a flexible doubly robust method to estimate the PTE of a high-dimensional surrogate in a non-randomized setting with implementation via the relaxed lasso and the super learner [7], 8]. Agniel & Parast [9] recently extended this approach to a longitudinal surrogate with a censored time-to-event outcome through the use of efficient influence functions for the treatment effect estimands, with implementation using a one-step plug-in estimator and a targeted minimum loss-based estimator [10].

These methods are useful for settings where treatment is not randomized and thus, one must account for individual characteristics which may be potential confounders. However, these methods do not offer a way to examine surrogate heterogeneity with respect to patient characteristics. Similar to (but different from) the idea of treatment effect heterogeneity, surrogate heterogeneity means that the surrogate may be useful, i.e., a valid replacement for the primary outcome, for some individuals but not others [11]. Of course, this is especially problematic if the surrogate is then used to replace the primary outcome in a future study, which is the ultimate goal of surrogate identification. Specifically, one may end up using a surrogate to make a decision about the effect of a treatment or exposure in a future study when in fact, the surrogate is a poor replacement of the primary outcome for the individuals in that study [12]. Recent work has offered methods to assess and test for surrogate heterogeneity, but they have been limited to randomized settings. For example, Roberts et al. [13] offered a Bayesian-based approach for surrogate validation conditional on baseline covariates in a principal stratification framework within a randomized setting. Also within a randomized setting, Knowlton et al. [14] and Parast et al. [11] proposed flexible approaches to estimate the PTE of a surrogate as a function of baseline covariates and formally test for evidence of heterogeneity.

To our knowledge, there do not exist any methods to assess heterogeneity in the PTE of a surrogate in a non-randomized setting. In this paper, we aim to fill this gap by proposing a framework to assess surrogate heterogeneity in non-randomized data and implement this framework using meta-learners. Our approach allows us to quantify surrogate strength and assess potential heterogeneity in surrogate strength with respect to patient characteristics while accommodating confounders through the use of flexible, off-the-shelf machine learning methods. In addition, we use our framework to identify covariate profiles where the surrogate is a valid replacement of the primary outcome, that is, covariate profiles in which the proportion of the treatment effect explained by the surrogate is greater than some prespecified threshold.

### 1.2 Organization of the paper

The paper is organized as follows. In Section 2 we describe our notation, setting, assumptions, and proposed framework. In Section 3 we propose general T-learner estimation methods and specifically consider two sets of base learners for illustration – simple linear models and generalized additive models (GAM), chosen to illustrate tradeoffs between flexibility and model specification. In Section 4 we propose a procedure to use our resulting estimates to identify covariate profiles where the surrogate is sufficiently strong. We examine the performance of our proposed methods using a simulation study in Section 5 and apply the methods to examine heterogeneity in the surrogacy of hemoglobin A1c as a surrogate for fasting plasma glucose in an observational data set in Section 6.

## 2 Setting and proposed framework

### 2.1 Notation, setting, and assumptions

Let *G* denote the treatment or exposure, where *G* = 1 indicates the treatment group and *G* = 0 indicates the control group, or a comparative treatment. Since the data are observational, treatment is *not* randomly assigned at baseline. Let **X** denote a *p* dimensional vector of baseline variables, *S* denote the surrogate marker measured after baseline, and *Y* denote the primary outcome of interest measured after baseline. Under the potential outcomes framework, we consider *S* ^(*g*)^ and *Y* ^(*g*)^, which denote the surrogate marker and primary outcome values under treatment *G* = *g*, respectively. The full potential data set thus encompasses (*Y* ^(1)^, *Y* ^(0)^, *S* ^(1)^, *S* ^(0)^, **X**), though we observe either (*Y* ^(1)^, *S* ^(1)^, **X**) or (*Y* ^(0)^, *S* ^(0)^, **X**) for each subject, contingent on the treatment received. Therefore, the observed data consist of independent and identically distributed (iid) copies of (*Y* ^(1)^, *S* ^(1)^, **X**) for the treatment group, denoted (*Y*_1*i*_, *S*_1*i*_, **X**_1*i*_) for *i* = 1, …, *n*_1_, and iid copies of (*Y* ^(0)^, *S* ^(0)^, **X**) for the control group, denoted (*Y*_0*i*_, *S*_0*i*_, **X**_0*i*_) for *i* = 1, …, *n*_0_. Here, *n*_*g*_ represents the number of individuals in treatment group *g*, and the total sample size is *n* = *n*_0_ + *n*_1_.

We first require a number of strong but common causal assumptions:

(C1) [Consistency] *Y* ^(*g*)^ = *Y* and *S* ^(*g*)^ = *S* when *G* = *g*;
(C2) [Positivity/Overlap] *P*{*π*_*g*_(**X**) > *ϵ*} = 1, where *π*_*g*_(**x**) = *P*(*G* = *g*|**X** = **x**), for some *ϵ* > 0, and *f*(*S*|**X** = **x**, *G* = *g*) > 0 for *g* = 0, 1;
(C3) [Unconfoundedness] *Y* ^(*g*)^, *S* ^(*g*)^ ⊥ *G*|**X** ;
(C4) *Y* ^(1)^ ⊥ *S* ^(0)^|*S* ^(1)^, **X** and *Y* ^(0)^ ⊥ *S* ^(1)^|*S* ^(0)^, **X**; and
(C5) *E*(*Y* ^(*g*)^|**X** = **x**) and *E*(*Y* ^(*g*)^|*S* ^(*g*)^, **X** = **x**) for *g* = 0, 1 are Lipschitz continuous.

Assumption (C1) states that the observed outcome and surrogate under treatment *g* are equal to their potential outcomes when treatment *G* = *g* is actually received. Assumption (C2) imposes overlap in two ways: overlap in treatment assignment/selection given baseline covariates **X**, and overlap in the conditional distribution of the surrogate *S* across treatment groups given **X**. Together, these conditions ensure that *μ*_1_(*s*, **x**) is well-defined on the support of *F*_*S*∣**X** = **x**,*G* = 0_. This is the minimal requirement needed for the integral *∫μ*_1_(*s*, **x**) *dF*_*S*∣**X** = **x**,*G* = 0_(*s*) to be well-defined. If these positivity conditions are violated, the estimand may no longer be identifiable without additional modeling assumptions or extrapolation beyond the observed support. Assumption (C3), commonly referred to as unconfoundedness, states that treatment assignment is independent of potential outcomes and potential surrogate values, conditional on observed covariates. In particular, this requires no unmeasured confounding between treatment assignment/selection and either the surrogate or the outcome. Assumption (C4) states that given the surrogate under one treatment assignment and the covariates, the outcome under that treatment is independent of the surrogate value under the other treatment. Lastly, Assumption (C5) is needed to ensure certain asymptotic properties, discussed in Section 3.3. While (C2) may be explored to some extent empirically, the other assumptions rely on potential outcomes that are not testable from observed data alone. Assumptions (C3) and (C4) in particular are strong assumptions that are difficult to verify; for example, (C4) involves potential outcomes under different treatments that are never simultaneously observed. Significant prior work has been done proposing methods to assess sensitivity to and quantify bias due to violations of such assumptions [15], [16], [17], [18]. In Appendix A we describe a sensitivity analysis examining violation of Assumption (C4) in our particular setting.

### 2.2 Proportion of treatment effect explained

In this paper, the measure of surrogate strength that we focus on is the proportion of the treatment effect on the primary outcome that is explained by the treatment effect on the surrogate marker, which is often abbreviated as PTE [19], 20]. The PTE is a single number summary defined based on contrasts between the overall treatment effect and the residual treatment effect after accounting for the effect on the surrogate. Here, we first describe this quantity as proposed in Wang & Taylor [19], which ignores potential heterogeneity and assumes randomized treatment. In the following section, we will build from this definition specifically incorporating heterogeneity and removing the randomization assumption. The overall treatment on *Y* is defined as

$$\Delta =E\left(Y^{\left(1\right)}-Y^{\left(0\right)}\right)$$

and the residual treatment effect is defined as

$$\Delta_{S}=\int E\left(Y^{\left(1\right)}-Y^{\left(0\right)}|S^{\left(1\right)}=S^{\left(0\right)}=s\right)dF_{S^{\left(0\right)}}\left(s\right),$$

where $F_{S^{\left(0\right)}}\left(\cdot \right)$ is the marginal cumulative distribution function of *S* ^(0)^. The residual treatment effect conceptually measures the treatment effect on the primary outcome that remains after adjusting for the treatment effect on the surrogate. Using these quantities, the proportion of the treatment effect explained is defined as *R*_*S*_ = (Δ − Δ_*S*_)/Δ = 1 − Δ_*S*_/Δ. Note that when Δ = 0, *R*_*S*_ is undefined. In general, high values of *R*_*S*_ indicate that a high proportion of the treatment effect is explained by *S* and thus, S is a strong surrogate, while lower values of *R*_*S*_ indicate a poor surrogate; we expand on this in Section 4.

### 2.3 Proposed framework to assess surrogate heterogeneity

Building from the standard PTE definition, we now define:

$$\begin{array}{ll} \Delta \left(x\right) & =E\left(Y^{\left(1\right)}|X=x\right)-E\left(Y^{\left(0\right)}|X=x\right),\;\text{and}\\ \Delta_{S}\left(x\right) & =\int E\left(Y^{\left(1\right)}-Y^{\left(0\right)}|S^{\left(1\right)}=S^{\left(0\right)}=s,X=x\right)dF_{S^{\left(0\right)}|X}\left(s\right), \end{array}$$

where $F_{S^{\left(0\right)|X}}\left(\cdot \right)$ is the conditional CDF of *S* ^(0)^|**X** = *x*, and we define the PTE as a function of **X** = **x**, so that *R*_*S*_(**x**) = 1 − Δ_*S*_(**x**)/Δ(**x**). Note that, similar to the unconditional quantities, if Δ(**x**) = 0 for a particular **x**, *R*_*S*_(**x**) is undefined.

We first consider Δ(**x**), which is the conditional average treatment effect (CATE). By Assumptions (C1)–(C3),

Δ(x)=E(Y∣X=x,G=1)−E(Y∣X=x,G=0),

which is identifiable from the available data. The problem of CATE estimation is a well-known problem and has received considerable attention in recent literature [21], [22], [23], [24], [25]. Classical nonparametric approaches to estimate CATE such as nearest neighbor matching or kernel methods suffer from the curse of dimensionality when the data have more than a couple of covariates, making them impractical for many modern applications. Particularly in our setting, the complexity of the covariates is a significant challenge since they may act both as confounders of treatment assignment and informative of the surrogate strength. Modern approaches that can accommodate higher covariate dimensions and maintain the flexibility of nonparametric approaches include “Meta-Learners”, for example, S-learners and T-learners [24], 25]. A meta-learner is simply the result of combining multiple “base learners”, which can be any supervised learning or regression estimators, in a specific way to estimate the quantity of interest, while allowing the base learners to take any form. So-called “S-learners” fit a Single learner that includes treatment assignment as a predictor, while “T-learners” instead fit separate learners for each treatment group, i.e., Two distinct learners. We focus here on T-learners due to their flexibility in capturing treatment effect heterogeneity without imposing structural assumptions about how treatment modifies the outcome-covariate relationship. To implement a T-learner for Δ(**x**), we require a decision for the base learner for the conditional expectation of the outcome given the covariates in each treatment group, which we denote as

$$\lambda_{g}\left(x\right)=E\left(Y^{\left(g\right)}|X=x\right)=E\left(Y|X=x,G=g\right),$$

where the second equality follows under Assumptions (C1)–(C3). Once the base learner is selected and used to estimate *λ*_*g*_(**x**), we denote the resulting estimates as ${\hat{\lambda }}_{0}\left(x\right)$ and ${\hat{\lambda }}_{1}\left(x\right)$ , and estimation of CATE is straightforward: $\hat{\Delta }\left(x\right)={\hat{\lambda }}_{1}\left(x\right)-{\hat{\lambda }}_{0}\left(x\right)$ .

Next, we consider estimation of the residual treatment effect Δ_*S*_(**w**), which is not as straightforward. Under Assumptions (C1)–(C4), we have

$$\begin{array}{ll} \Delta_{S}\left(x\right) & =\int E\left(Y|S=s,X=x,G=1\right)dF_{S|X,G=0}\left(s\right)\\ & \;-\int E\left(Y|S=s,X=x,G=0\right)dF_{S|X,G=0}\left(s\right)\\ & =\int \mu_{1}\left(s,x\right)dF_{S|X,G=0}\left(s\right)-\int \mu_{0}\left(s,x\right)dF_{S|X,G=0}\left(s\right), \end{array}$$

where *μ*_*g*_(*s*, **x**) = *E*(*Y* ^(*g*)^∣*S* ^(*g*)^ = *s*, **X** = **x**) represents the conditional mean function, and where *F*_*S*|**X**,*G* = 0_(⋅) represents the cumulative distribution function of *S* ^(0)^ given **X** = **x**. Similar to Δ(**x**), we propose to implement a T-learner for Δ_*S*_(**x**), but we now require a selection of base learners for both *μ*_*g*_(*s*, **x**) and *ζ*_0_(**x**) = *E*(*S* ^(0)^∣**X** = **x**).

In the following section, we use sample-splitting to obtain these estimates and provide details for the implementation of both T-learners to obtain estimates of Δ(**x**), Δ_*S*_(**x**), and *R*_*S*_(**x**) using two sets of base learners for illustration: a linear model and a generalized additive model (GAM).

**Remark.**

Note that the construction of *R*_*S*_(**x**) does not inherently impose the constraint that *R*_*S*_(**x**) ∈ [0, 1], meaning there is no requirement that 0 ≤ Δ_*S*_(**x**) ≤ Δ(**x**). This issue has been explored in greater detail in other works, such as Stijven et al. [26] in the surrogate setting, where it is argued that values exceeding 1 can still be meaningful, and more broadly in Preacher & Kelley [27], which examines methods for decomposing effects into direct and indirect components. In fact, the PTE can extend beyond the [0,1] range unless additional constraints are imposed. One sufficient set of assumptions that ensures *R*_*S*_(**x**) ∈ [0, 1] aligns with those preventing the surrogate paradox, which is discussed in more detail in Section 7. However, we do not explicitly impose these assumptions here and, therefore, do not require *R*_*S*_(**x**) to remain strictly within the unit interval.

## 3 Implementation and inference

### 3.1 Implementation via metalearners

Implementation of our framework requires selecting base learners for the following components: the outcome models *λ*_*g*_(**x**), the outcome-surrogate conditional models *μ*_*g*_(*s*, **x**), and the surrogate model in the control group *ζ*_0_(**x**). Many reasonable choices exist for estimating these components, from simple, computationally efficient models to more complex, flexible models. We focus on using two sets of base learners – a linear model and a GAM – via the following algorithm.

Algorithm for Estimating *R*_*S*_ ( **x** )

**Step 1: Split the Data.** Split the available data set into a training set that will be used to build the base learners for *λ*_*g*_(**x**), *μ*_*g*_(*s*, **x**), and *ζ*_0_(**x**), and a testing set that will be used to obtain predictions from the trained learners and estimate Δ(**x**), Δ_*S*_(**x**), and *R*_*S*_(**x**). Use of sample-splitting aims to prevent overfitting and ensure honest assessment of the method’s performance with respect to the estimate’s bias and variance.

**Step 2: Select a Base Learner.** Choose a supervised learning method (e.g., linear model, GAM).

**Step 3: Estimate the Conditional Average Treatment Effect,** Δ(**x**).

(a) *Fit Learners for Each Group (T-Learner)*: Using the selected learner, build a learner using the training set for *λ*_*g*_(**x**) = *E*(*Y*∣**X** = **x**, *G* = *g*), for *g* = 0, 1.
(b) *Predict* *λ*_*g*_(**x**): Obtain predictions ${\hat{\lambda }}_{0}\left(x\right)$ and ${\hat{\lambda }}_{1}\left(x\right)$ from the fitted learners using the testing set.
(c) *Estimate* Δ(**x**): Compute the CATE as: $\hat{\Delta }\left(x\right)={\hat{\lambda }}_{1}\left(x\right)-{\hat{\lambda }}_{0}\left(x\right)$ .

**Step 4: Estimate the Residual Treatment Effect,** Δ_*S*_(**x**).

(a) *Fit Learners*: Using the selected learner, build learners using the training set for *μ*_0_(*s*, **x**), *μ*_1_(*s*, **x**), and *ζ*_0_(**x**).
(b) *Predict* ${\hat{\zeta }}_{0}\left(x\right)$ : For each **x** in the testing set, predict ${\hat{\zeta }}_{0}\left(x\right)$ , the expected value of *S* ^(0)^ given **X** = **x**.
(c) *Evaluate* ${\hat{\mu }}_{g}\left(s,x\right)$ *at* ${\hat{\zeta }}_{0}\left(x\right)$ : Using a plug-in estimator, use the testing set to predict ${\hat{\mu }}_{1}({\hat{\zeta }}_{0}\left(x\right),x)$ and ${\hat{\mu }}_{0}({\hat{\zeta }}_{0}\left(x\right),x)$ .
(d) *Estimate* Δ_*S*_(**x**): Compute the residual treatment effect as:
(1) $${\hat{\Delta }}_{S}\left(x\right)={\hat{\mu }}_{1}({\hat{\zeta }}_{0}\left(x\right),x)-{\hat{\mu }}_{0}({\hat{\zeta }}_{0}\left(x\right),x),$$

**Step 5: Estimate the Proportion of Treatment Effect Explained,** *R*_*S*_(**x**). Using the estimates of Δ(**x**) and Δ_*S*_(**x**), calculate: ${\hat{R}}_{S}\left(x\right)=1-{\hat{\Delta }}_{S}\left(x\right)/\hat{\Delta }\left(x\right)$ . As noted earlier, if Δ(**x**) = 0 for a particular **x**, *R*_*S*_(**x**) is undefined; with respect to the estimated quantities, when the estimate of Δ(**x**) is close to 0, it is expected that corresponding estimates of *R*_*S*_(**x**) may be unstable.

We fit both learners in R, using the standard lm() function available in base R for the linear base learners and the gam() function from the mgcv library for the GAM learners. The gam() function represents the smooth functions of the specified covariates using penalized regression splines and selects the optimal basis functions for these splines via generalized cross-validation for smoothing parameter estimation [28], 29].

For variance estimation, we used the nonparametric bootstrap with 500 iterations, obtaining all (1 − *α*)% confidence intervals for Δ(**x**), Δ_*S*_(**x**), and *R*_*S*_(**x**) as the *α*/2 and 1 − *α*/2 percentiles of the bootstrap distributions. This approach does not rely on a normal approximation (i.e., an estimate ± 1.96 times a standard error), which would require asymptotic normality. Instead, the bootstrap directly approximates the sampling distribution of each estimator. Formal justification of this procedure requires that the bootstrap distribution converges to the true sampling distribution. For fixed-dimensional regression models such as linear models and GAMs, this follows from established asymptotic theory, and both delta-method and bootstrap procedures are valid.

### 3.2 Alternative approaches

There are two particularly notable aspects of our algorithm within Step 4. The first is that we use the fitted learner ${\hat{\zeta }}_{0}\left(\cdot \right)$ to predict *S*_0*i*_ given **X**_*i*_ = **x** for every observation *i* in the test set, including those for whom we have observed *S*_0*i*_, i.e., those in the control group. We do this to ensure consistency in estimation and mitigate noise from individual observations. With respect to consistency, applying ${\hat{\zeta }}_{0}\left(\cdot \right)$ uniformly across both treated and control groups ensures that the predicted *S*_0*i*_ are defined in the same way for every unit, rather than mixing raw outcomes for some units and model-based predictions for others. Noise is reduced because observed *S*_0*i*_ values may reflect substantial idiosyncratic variation, whereas the predictions ${\hat{\zeta }}_{0}\left(x\right)$ pool information across subjects and smooth out individual-level fluctuations. However, one may alternatively consider using ${\hat{\zeta }}_{0}\left(\cdot \right)$ to predict *S*_0*i*_ only for the treated group (*G* = 1), while directly using the observed *S*_0*i*_ for the control group (*G* = 0). This approach avoids unnecessary estimation error in cases where *S*_0*i*_ is observed without noise and the model may introduce bias. If the learner for *ζ*_0_(**x**) is misspecified, replacing true observations with predictions in *G* = 0 could reduce the accuracy of subsequent estimations. Thus, a reasonable diagnostic would be to compare the distributions of the observed *S*_0*i*_ and the predicted ${\hat{\zeta }}_{0}\left(x\right)$ in the control group. If these distributions align closely, using ${\hat{\zeta }}_{0}\left(x\right)$ for everyone in both groups is unlikely to introduce significant bias. If they differ substantially, then directly using observed values for *G* = 0 may be preferable. In our simulation study, described in Section 5, we compared these distributions and found them to be quite similar; as a sensitivity analysis, we additionally carried out simulations where raw observed values of *S*_0*i*_ were used in *G* = 0 rather than predictions, and the methods performed slightly worse in terms of bias (not shown), which supports our decision to use predictions for all units, at least in these settings.

The second aspect of our approach is the use of a plug-in estimator ${\hat{\mu }}_{g}\left({\hat{\zeta }}_{0}\left(x\right),x\right)$ as an approximation to the integral

$$\int \mu_{g}\left(s,x\right)\;dF_{S|X=x,G=0}\left(s\right).$$

Rather than explicitly estimating the conditional distribution *F*_*S*∣**X**,*G* = 0_ and performing numerical integration, we approximate the integral by evaluating *μ*_*g*_(*s*, **x**) at *ζ*_0_(**x**) = *E*(*S* ^(0)^∣**X** = **x**). This choice is motivated by both computational and statistical considerations. While numerical integration is feasible, any such integration strategy requires estimation of the conditional distribution *F*_*S*∣**X**,*G* = 0_ (or its density), which is known to be a difficult problem without strong parametric assumptions, particularly when **X** is moderate- or high-dimensional. These challenges are further amplified when combined with bootstrap-based inference. In contrast, the plug-in estimator yields a smooth, low-variance approximation that scales naturally with flexible supervised learning methods. We discuss the validity of this approximation in Section 3.3. In addition, Appendix B reports simulation results comparing our proposed approach to a Monte Carlo approach that evaluates the integral over the conditional distribution of *S* ^(0)^, demonstrating that the plug-in approximation exhibits similar finite-sample behavior and reduced computational cost in the settings we consider.

### 3.3 Asymptotic properties

In this section, we consider the statistical properties of the proposed estimators, focusing on consistency and asymptotic behavior of our two base learners: linear models and GAMs. For each learner, we demonstrate the consistency of $\hat{\Delta }\left(x\right)$ and ${\hat{\Delta }}_{S}\left(x\right)$ ; it will then follow from these properties that ${\hat{R}}_{S}\left(x\right)$ is a consistent estimator of *R*_*S*_(**x**).

We first discuss consistency of ${\hat{\lambda }}_{0}\left(x\right)$ and ${\hat{\lambda }}_{1}\left(x\right)$ , for each base learner under assumptions (C1)–(C5). When these components are consistent, it follows that $\hat{\Delta }\left(x\right)$ is a consistent estimate of Δ(**x**). When the base learners are linear models and the linear models are correctly specified, consistency of ${\hat{\lambda }}_{0}\left(x\right)$ and ${\hat{\lambda }}_{1}\left(x\right)$ follow from classical properties of ordinary least squares (OLS) regression. When the base learners are GAMs and the additive effects are correctly specified, consistency of the estimators as implemented here in the mgcv package has been shown in prior work [30], [31], [32], [33] under appropriate smoothness and regularization conditions.

Regarding ${\hat{\Delta }}_{S}\left(x\right)$ , the consistency of each component *μ*_0_(*s*, **x**), *μ*_1_(*s*, **x**), and *ζ*_0_(**x**) follow from the previous paragraph with the various learners. The more delicate aspect is the validity of our use of the plug-in estimator ${\hat{\mu }}_{g}\left({\hat{\zeta }}_{0}\left(x\right),x\right)$ as an approximation to the integral *∫μ*_*g*_(*s*, **x**)*dF*_*S*|**X**,*G* = 0_(*s*), ensuring that ${\hat{\mu }}_{g}\left({\hat{\zeta }}_{0}\left(x\right),x\right)$ is a consistent estimator of the integral. Assumption (C5) is needed tp support validity of this approximation as it ensures that small perturbations in *s* lead to controlled deviations in *μ*_*g*_(*s*, **x**) and therefore that *μ*_*g*_(*ζ*_0_(**x**), **x**) is a good approximation to *E*(*μ*_*g*_(*S*, **x**)|**X**) [34]. In addition to (C5), we require:

(C6) Var(*S* ^(0)^∣**X** = **x**) → 0 uniformly in **x** as the sample size increases.

Under both conditions, a second-order Taylor expansion of *μ*_*g*_(*s*, **x**) around *s* = *ζ*_0_(**x**) shows that the approximation error produced by replacing the integral with *μ*_*g*_(*ζ*_0_(**x**), **x**) vanishes asymptotically [35], yielding consistency of ${\hat{\Delta }}_{S}\left(x\right)$ for Δ_*S*_(**x**). Specifically, consider expanding *μ*_*g*_(*s*, **x**) around *ζ*_0_(**x**) = *E*[*S* ^(0)^∣**X** = **x**] as follows:

$$\mu_{g}\left(S^{\left(0\right)},x\right)=\mu_{g}\left(\zeta_{0}\left(x\right),x\right)+\mu_{g}^{\prime }\left(\zeta_{0}\left(x\right),x\right)\left(S^{\left(0\right)}-\zeta_{0}\left(x\right)\right)+\frac{1}{2}\mu_{g}^{\prime \prime }\left(\tilde{S},x\right){\left(S^{\left(0\right)}-\zeta_{0}\left(x\right)\right)}^{2},$$

where $\tilde{S}$ lies between *S* ^(0)^ and *ζ*_0_(**x**). Taking the expectation over *S* ^(0)^∣**X** = **x** gives

$$\int \mu_{g}\left(s,x\right)\;dF_{S|X=x,G=0}\left(s\right)=\mu_{g}\left(\zeta_{0}\left(x\right),x\right)+\frac{1}{2}E\left[\mu_{g}^{\prime \prime }\left(\tilde{S},x\right)\left.{(S^{\left(0\right)}-\zeta_{0}\left(x\right))}^{2}\right|X=x\right],$$

where the linear term vanishes since $E\left[S^{\left(0\right)}-\zeta_{0}\left(x\right)|X=x\right]=0$ . Assuming a standard regularity condition that the second derivative $\mu_{g}^{\prime \prime }\left(s,x\right)$ is bounded, the remainder term is of order *O*(Var(*S* ^(0)^∣**X** = **x**)). Therefore, as Var(*S* ^(0)^∣**X** = **x**) → 0, the plug-in estimator satisfies

$$\Delta_{S}\left(x\right)\approx \mu_{1}\left(\zeta_{0}\left(x\right),x\right)-\mu_{0}\left(\zeta_{0}\left(x\right),x\right)=\Delta_{S}^{\text{plug}-\text{in}}\left(x\right),$$

and the approximation error vanishes asymptotically. With consistent estimators ${\hat{\mu }}_{g}$ and ${\hat{\zeta }}_{0}$ , it follows that ${\hat{\Delta }}_{S}\left(x\right)$ is a consistent estimator of Δ_*S*_(**x**). When Var(*S* ^(0)^∣**X**) does not vanish, the plug-in estimator may exhibit asymptotic bias. In such settings, alternative methods may be preferable. Appendix B presents simulation results using a Monte Carlo approach in place of the plug-in approximation.

## 4 Covariate profile identification

In the previous section, we introduced meta-learners to estimate *R*_*S*_(**x**), which quantifies the strength of the surrogate as a function of **x**. Here, we leverage these estimates to identify covariate profiles where the surrogate is sufficiently strong to replace the primary outcome. After all, the ultimate goal of investigating surrogacy and heterogeneity is to guide the effective and appropriate use of surrogate markers in a future study.

Recall that higher values of *R*_*S*_(**x**) indicate stronger surrogacy. Though there is no established threshold for what value reflects a valid surrogate, previous work has often considered a surrogate to be “strong” if this value or the lower bound of its confidence interval exceeds 0.50 or 0.75 [36], 37]. We denote this threshold as *κ*. Ideally, *κ* should be selected *a priori*, informed by domain expertise and study-specific considerations. However, it may also be treated as a tunable parameter when factoring in cost-effectiveness, a topic we discuss further in Section 7.

Given a chosen *κ* and a specific x* covariate profile, our goal is to determine whether the surrogate is sufficiently strong by testing the following null hypothesis:

$$H_{0}:R_{S}\left(x^{*}\right)\leq \kappa .$$

We consider testing *H*_0_ by constructing a one-sided (1 − *α*)% confidence interval for *R*_*S*_(x*) using our bootstrap samples and rejecting *H*_0_ if *κ* is less than the lower bound of the interval. More specifically, we calculate

$$p^{*}=\frac{1}{B}\overset{B}{\underset{b=1}{\sum }}I\left(R_{S}^{\left(b\right)}\left(x^{*}\right)\leq \kappa \right),$$

where $R_{S}^{\left(b\right)}\left(x^{*}\right)$ is the bootstrapped estimate of *R*_*S*_(x*) from the *b*-th bootstrap sample, *B* is the number of bootstrapping iterations (*B* = 500 in our simulation study), and *I* is the indicator function. To account for multiple testing, we additionally apply the Benjamini-Hochberg procedure to all calculated p*’s (if more than one) and conclude that the surrogate is sufficiently strong for covariate profile x* if the adjusted p* is less than *α* [38].

We investigate the performance of this identification approach in Section 5, in settings where the true PTE is known, by examining the positive predictive value (PPV), negative predictive value (NPV), specificity, and sensitivity of the testing results.

## 5 Simulation study

### 5.1 Simulation goals and setup

We conducted a simulation study to evaluate the performance of our proposed methods across multiple settings. We considered three primary settings of increasing complexity in their data generating processes. Setting 1 featured a linear data generating process, with the true PTE, *R*_*S*_(**x**), ranging from 0.32 to 0.65, and where linear models were expected to perform well. Setting 2 introduced nonlinear components to the data generating process but remained additive in nature (*R*_*S*_(**x**) ranging from 0.14 to 0.64), making it particularly suitable for the GAM base learners. Setting 3 incorporated more complex relationships (*R*_*S*_(**x**) ranging from 0.14 to 0.64) that violated the additive assumption of GAMs. To reflect a non-randomized setting, in all simulation settings, the treatment assignment *G* was dependent on the baseline covariates and was constructed such that the treatment group sizes were approximately equal. Details of the simulation settings are provided in Appendix C, along with an additional Setting 4 featuring no heterogeneity (that is, *R*_*S*_(**x**) = 0.67 for all **x**). R code to reproduce all simulation results is available at: https://github.com/rebeccaknowlton/obshetsurr-simulations.

All settings had a sample size of *n* = 2,000, a test set of 200 randomly selected individuals, and six baseline covariates comprising **X**. In Settings 1–3, there was heterogeneity in surrogacy with respect to the first covariate, *X*_1_; PTE was constant with respect to the other baseline covariates. Bootstrapped estimates with 500 iterations were used for standard error estimation and confidence interval construction. For the purpose of covariate profile identification as described in Section 4, we used a threshold of *κ* = 0.5. All simulation results were summarized across 500 iterations, and performance was summarized in terms of median absolute bias, the empirical median absolute deviation (MAD) which is a robust measure of spread that is less sensitive to outliers and heavy tails than the standard deviation [39], the median of the bootstrap estimates of MAD (labeled MAD^*b*^), median squared error, and confidence interval coverage of the true *R*_*S*_(**x**).

### 5.2 Simulation results

**Figure 1:**
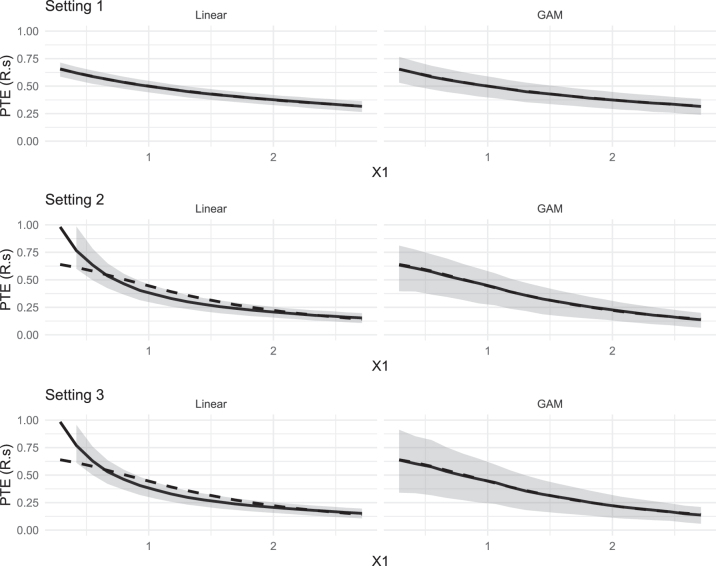
Estimated *R*_*S*_(**x**) (solid lines) versus true *R*_*S*_(**x**) (dashed lines) plotted against *X*_1_, the baseline covariate featuring heterogeneous surrogate strength in our simulations, with pointwise confidence bands (grey shading) obtained using bootstrapping.

Figure 1 displays the resulting estimates (solid line) and confidence intervals (gray shading) for *R*_*S*_(**x**), plotted against the truth (dashed line), for Settings 1–3 featuring the linear and GAM base learners. The figure shows that the approach using linear base learners perform exceptionally well in Setting 1 as expected, with very little bias and low variance compared to the GAM learners. However, the linear base learners produce biased results for some ranges of *X*_1_ in Settings 2 and 3, when the true data generating process is not linear. Interestingly, the GAM base learners perform quite well not only in Setting 2 (which we would expect), but also in Setting 3 when the data generating process was not additive.

The overall results of our simulation settings are summarized numerically in Table 1. These results are averaged over the grid of *X*_1_. Again, Setting 1 showcases the strong performance of the linear base learners when appropriate, with low bias, small standard errors, and coverage reasonably close to the nominal 95 % confidence level. In Settings 2 and 3, when the linear models do not hold, coverage deteriorates due to the estimates being biased in some regions of the covariate space. Meanwhile, the GAM base learners continue to perform well in terms of high coverage and low MSE. Even though the absolute bias is somewhat higher in Setting 3 when the assumptions of the GAM are violated, the model still performs quite well overall. Throughout settings and choice of base learners, MAD^*b*^ estimated via resampling is reasonably close to the MAD.

**Table 1:** Simulation results for *R*_*S*_(**x**) in Settings 1–3, averaged over *X*_1_, where Bias reflects the absolute value of the difference between the estimate and the truth, summarized as the median of 500 iterations; MAD reflects the empirical median absolute deviation (calculated as the median absolute deviation of estimates across the 500 simulation iterations, using the mad() function in R); MAD^*b*^ reflects the median of the bootstrap estimates of MAD; MSE represents the median squared error; Coverage indicates the coverage rate of 95 % bootstrap confidence intervals with respect to the truth.

	Setting 1	
	Linear	GAM
Bias	0.015	0.021
MAD	0.023	0.031
MAD^*b*^	0.026	0.044
MSE	0.000	0.000
Coverage	0.970	0.984

	**Setting 2**	
	**Linear**	**GAM**
Bias	0.057	0.033
MAD	0.037	0.050
MAD^*b*^	0.043	0.061
MSE	0.008	0.001
Coverage	0.775	0.972

	**Setting 3**	
	**Linear**	**GAM**
Bias	0.058	0.041
MAD	0.035	0.062
MAD^*b*^	0.040	0.075
MSE	0.009	0.002
Coverage	0.752	0.970

To evaluate the covariate profile identification procedure described in Section 4, we use our proposed approach to identify covariate profiles in the testing set as those where the surrogate is strong $\left({\hat{R}}_{S}\left(x^{*}\right)>\kappa \right)$ , and we compare to the truth i.e., $\left(R_{S}\left(x^{*}\right)>\kappa \right)$ , where *κ* = 0.5 in all settings. The performance is summarized in terms of positive predictive value (PPV), negative predictive value (NPV), specificity, and sensitivity in Table 2. Across all settings and both base learners, we see strong performance in terms of PPV and specificity (note that higher values indicate better performance for all four quantities). The NPV is reasonably high, ranging from 0.727 to 0.838 across settings and both learners. In contrast, sensitivity is somewhat low, meaning that among covariate profiles where the surrogate is truly strong, the methods struggle to correctly identify a high proportion of them as such. For the linear base learners, it is worth noting (as seen in Figure 1) that the bias in Settings 2 and 3 is a result of estimating the PTE to be *higher* than the truth, and thus even in these settings, the linear base learners are reasonably successful at covariate profile identification. Of course, this is particular to this simulation setting and is not expected to hold generally for linear base learners. Compared to the linear base learners, sensitivity is lower for the GAM-based approach, suggesting that the identification procedure can be quite conservative. These results, along with Table 1, demonstrate an expected tradeoff in that more flexible learners such as GAMs yield better coverage but lower sensitivity. Importantly, the threshold-based performance metrics in Table 2 are particularly sensitive to high variance in the estimates such that fluctuations around the 0.5 cutoff can lead to large swings in classification, even if the underlying estimator is unbiased on average. In contrast, the linear learner, although biased in Settings 2 and 3, produces smoother and lower-variance estimates, which can make its threshold-based performance appear better. Notably, in practice, it is likely preferable to be more conservative in identifying covariate profiles where it is appropriate to substitute the surrogate rather than less conservative.

**Table 2:** Performance assessment for the covariate profile identification procedure in Settings 1–3 and summarized over 500 iterations. PPV reflects the positive predictive value, i.e., the proportion of covariate profiles identified by our procedure as those where the surrogate is strong, where the surrogate is truly strong; NPV reflects the negative predictive value, i.e., the proportion of covariate profiles identified by our procedure as those where the surrogate is weak, where the surrogate is truly weak; Specificity reflects the proportion of covariate profilesin which the surrogate is truly weak (*R*_*S*_(**x**) < = *κ*) that have been correctly identified as such; Sensitivity reflects the proportion of covariate profiles in which the surrogate is truly strong (*R*_*S*_(**x**) > *κ*) that have been correctly identified.

	Setting 1	
	Linear	GAM
PPV	0.998	0.998
NPV	0.828	0.727
Specificity	0.999	1.000
Sensitivity	0.584	0.251
	**Setting 2**	
	**Linear**	**GAM**
PPV	1.000	0.990
NPV	0.830	0.736
Specificity	1.000	1.000
Sensitivity	0.451	0.036
	**Setting 3**	
	**Linear**	**GAM**
PPV	1.000	0.981
NPV	0.838	0.732
Specificity	1.000	1.000
Sensitivity	0.481	0.019

Overall, these results demonstrate reasonable performance of the proposed methods in various settings in terms of both estimation of *R*_*S*_(**x**) and covariate profile identification. R code to reproduce these simulation results is available at: https://github.com/rebeccaknowlton/obshetsurr-simulations.

## 6 Example

We illustrate our proposed framework using data from the National Health and Nutrition Examination Survey (NHANES), which is a routine national survey administered by the United States Centers for Disease Control and Prevention (CDC) National Center for Health Statistics [40]. NHANES aims to measure the health and nutrition of adults and children in the United States and includes health exams and laboratory work.

We focus on examining the difference in fasting plasma glucose levels between obese and non-obese individuals, where obesity is defined as a body mass index (BMI) of 30 or greater. In this context, fasting plasma glucose serves as the primary outcome of interest, while the treatment/exposure is obesity status, classified as obese (treated) versus non-obese (control). Numerous studies have established a strong association between obesity and elevated fasting plasma glucose levels, a critical indicator of metabolic health and a key risk factor for serious health conditions [41], [42], [43], [44]. As a potential surrogate marker, we consider hemoglobin A1c (HbA1c), a biomarker that reflects long-term glucose regulation. Unlike fasting plasma glucose, HbA1c does not require fasting before laboratory testing, making it more convenient to measure and reducing participant burden. Our proposed framework is particularly relevant in this setting, as obesity is not randomly assigned – a key assumption underlying many traditional methods for surrogate marker evaluation. The baseline covariates, **X**, for this illustration are age, sex, and (total) cholesterol. Therefore, our overall goal is to use our framework to examine the potential heterogeneity (with respect to age, sex, and cholesterol) in surrogate strength when considering HbA1c as a surrogate for fasting plasma glucose when comparing obese to non-obese individuals.

**Figure 2:**
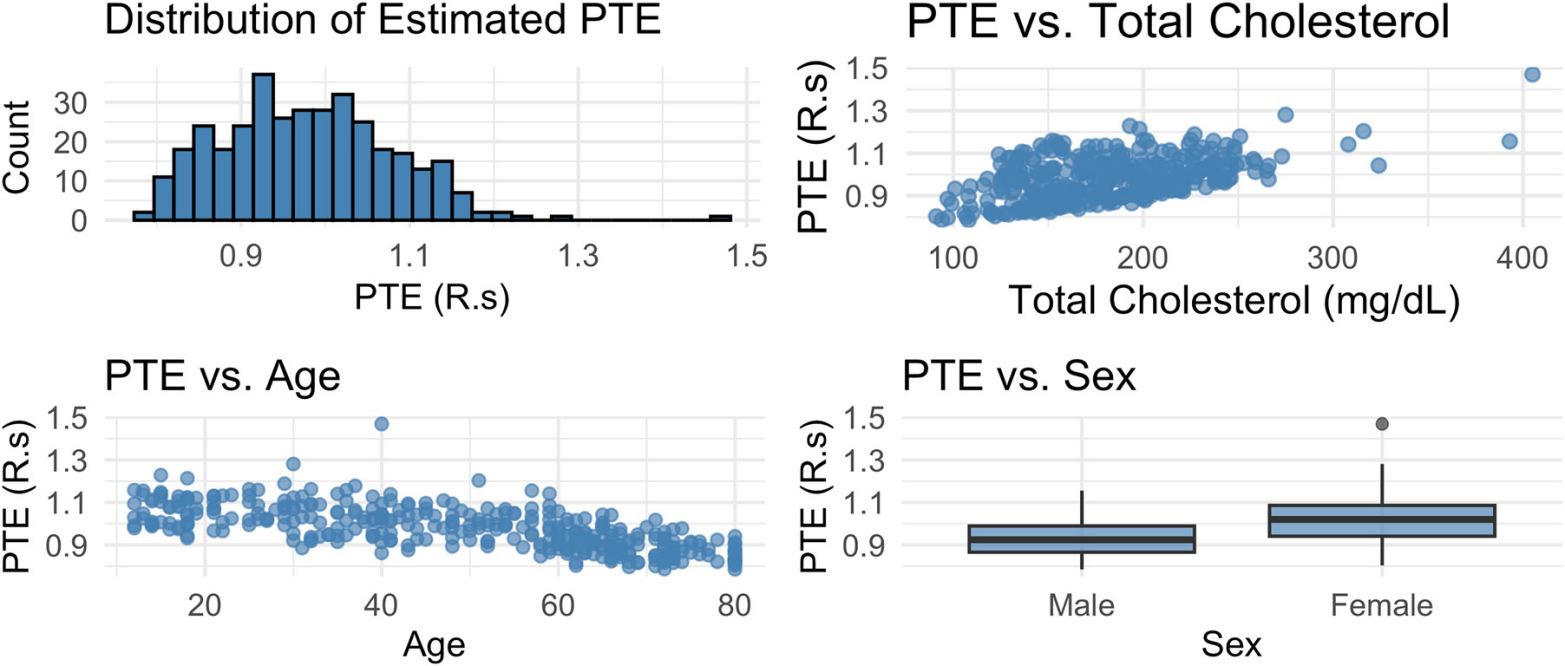
Estimation results for the NHANES survey data, evaluating the strength of HbA1c as a surrogate marker for plasma fasting glucose, when the exposure is obesity status; subfigures show the distribution of PTE estimates (top left panel) and PTE estimates by cholesterol (top right), age (bottom left), and sex (bottom right).

We use cross-sectional survey data from the 2-year cycle August 2021–August 2023, including adults and children, which are publicly available on the CDC’s website. Individuals missing fasting plasma glucose, HbA1c, BMI, age, sex, and cholesterol were excluded. Our final analytic sample size was *n* = 3,476, with *n*_0_ = 2,158 non-obese individuals and *n*_1_ = 1,318 obese individuals. We split our sample, retaining 350 observations for testing data (about 10 % of the total sample size, similar to our numerical studies). In practice, applying our framework requires selecting appropriate base learners, which will naturally be context dependent. For this illustration, we demonstrate linear models as the base learners; in Appendix D, we additionally include results using GAMs as the base learners. We used our approach to obtain PTE estimates for the testing data set, the results of which are displayed in Figure 2. Recall that, as discussed in Section 2.3, *R*_*S*_(**x**) may be larger than 1. In the top left panel, we show the distribution of PTE estimates which demonstrates that the estimated PTE is generally high, i.e., HbA1c appears to be a good surrogate for plasma fasting glucose in this data set. The remaining panels show PTE estimates by cholesterol (top right), age (bottom left), and sex (bottom right), indicating that the surrogate strength varies across these different baseline characteristics, with higher estimated PTE for individuals with higher cholesterol, younger ages, and for females.

To demonstrate the practical application of our estimates for a future study, we consider six covariate profiles, as shown in Table 3. Leveraging the trained models from the NHANES data, we compute 95 % confidence intervals for the covariate profile PTE, illustrating how surrogate strength varies based on covariates. As a potential application in future studies, one might deem the surrogate marker a suitable substitute if the lower bound of the confidence interval is at least 0.70. Under this criterion, the surrogate alone would suffice for covariate profiles 2, 3, 4, and 6, whereas the primary outcome would still need to be measured for covariate profiles 1 and 5. These findings highlight the ability of our approach to incorporate covariate information, facilitating more tailored decisions about future outcome measurement in studies involving non-randomized exposures. R code to reproduce results is available at: https://github.com/rebeccaknowlton/obshetsurr-NHANES-example.

**Table 3:** Estimated 95 % confidence intervals for the PTE for six covariate profiles defined by age, sex, and cholesterol levels using the proposed method applied to the NHANES survey data.

Covariate profile	Age	Sex	Cholesterol (mg/dL)	Estimated 95 % confidence interval for PTE
1	65	Male	160	(0.68, 1.10)
2	45	Female	220	(0.86, 1.46)
3	35	Female	250	(0.74, 3.29)
4	50	Male	180	(0.75, 1.15)
5	70	Male	140	(0.63, 1.12)
6	30	Female	210	(0.85, 1.78)

## 7 Discussion

We have proposed a framework for evaluating heterogeneous surrogate strength in observational settings characterized by complex covariate relationships. Our methodology allows flexibility in the choice of base learners within the T-Learner, ranging from computationally efficient linear models to more flexible GAMs. Between the two sets of learners we examined, the GAM offered the most consistent advantages, outperforming the linear model in two of the three simulation settings. Our covariate-specific approach to evaluating surrogate validity aligns with the growing emphasis on personalized decision-making, especially in contexts involving complex and heterogeneous data [45], 46]. Rather than relying on a rigid, one-size-fits-all decision rule, our framework enables robust, data-driven decisions tailored to specific individual characteristics. In addition, we developed appropriate statistical tests for evaluating surrogate strength as measured using clinically relevant thresholds and validated the performance of our methods through simulation studies. An R package implementing our methods, cohetsurr, is available on CRAN [47].

While our numerical studies specifically examine linear models and GAMs as base learners within our general framework, many other learners could be incorporated. An appealing direction is the use of the super learner, which adaptively combines multiple learners to improve predictive accuracy and robustness [8]. Beyond expanding the choice of base learners, one could also move beyond pure outcome modeling to approaches that integrate both outcome regression and inverse propensity score weighting. Such doubly robust strategies, as considered in Han et al. [5] and Kennedy [48], can offer protection against model misspecification while also achieving efficiency gains. Notably, for these alternative learners, as well as for the learners examined here, cross-fitting could be used to improve finite-sample efficiency. We instead adopt a single split to enforce a clear separation between discovery and evaluation, prioritizing robustness and interpretability in the presence of potentially extensive heterogeneity exploration. In addition, though our method can accommodate heterogeneity with respect to multiple baseline covariates, our numerical studies focus on settings where heterogeneity is induced by one baseline covariate; future work examining performance in higher dimensional setting is warranted.

Our framework notably shares mathematical similarities with the topic of moderated mediation; however, there are fundamental conceptual differences in the objectives that are worth discussing [26], 49]. In general, surrogate evaluation and mediation analysis, which involves nested counterfactuals, are only equivalent under additional untestable assumptions. Moderated mediation focuses on understanding the mechanism through which a treatment affects an outcome and how this mechanism varies across subgroups [50], 51]. In contrast, our approach to surrogate markers is not necessarily concerned with establishing causal mechanisms, but rather with identifying variables that reliably capture the treatment effect and thus can substitute for the primary outcome in future studies. This distinction is crucial: while mediation analysis seeks to decompose and explain causal pathways, surrogate evaluation in the PTE framework aims to validate replacement outcomes that capture treatment effects, regardless of the underlying mechanisms. Recent methodological advances in heterogeneous mediation effects, such as the Bayesian tree ensemble approach in [52], share our interest in effect heterogeneity but differ fundamentally in their goal of understanding mechanistic pathways rather than outcome substitution. Our framework therefore complements rather than overlaps with these developments.

This proposed approach relies on causal assumptions that, while common in the literature, may not hold in practice. Furthermore, more assumptions may be needed if one is interested in ensuring *R*_*S*_(**x**) ∈ [0, 1] and guarding against the surrogate paradox – a phenomenon where a positive treatment effect on the surrogate and positive surrogate-outcome association paradoxically coexist with a negative treatment effect on the primary outcome. Protection against this paradox typically requires additional assumptions: monotonicity in the surrogate-outcome relationship, a non-negative treatment effect on the surrogate, and non-negative direct treatment effects conditional on the surrogate and baseline characteristics [49], 53], 54]. While these conditions are important when using surrogates as outcome replacements in future studies, they are less critical in our context where we focus on evaluating surrogate strength in a single study where the primary outcome is also observed. Still, researchers applying our methods should consider whether such additional assumptions might be warranted for their specific application, particularly if the findings will inform future surrogate-based studies.

With respect to the statistical properties of our proposed methods, it is important to consider the convergence rates of the various estimators. The T-Learner approach, while offering flexibility, faces inherent challenges in estimation efficiency and, in fact, often perform poorly when the true heterogeneity is simple, or when the treatment groups are very different sizes [24], 25]. By fitting separate models for the treatment and control groups, we effectively reduce the available sample size for each model, potentially slowing convergence even when we have consistency. This challenge is further compounded by our sample-splitting procedure. The convergence concerns are especially pronounced when using machine learning methods, which typically require substantial data to achieve reliable estimates. While simpler base learners like linear models offer faster convergence rates under limited data scenarios, they may be biased when the true underlying relationships are complex. This creates a practical trade-off between bias and variance that must be carefully considered when choosing a base learner, depending on the sample size and complexity of the data.

Our framework enables covariate-specific estimation of the PTE and identification of covariate profiles for which the PTE is high in non-randomized settings, raising an important question about how such information should be used in practice. When PTE heterogeneity is present, the central question is not simply whether a surrogate is valid on average, but if and when the surrogate can replace the primary outcome. If our methods indicate that the PTE is heterogeneous but uniformly high across all covariate profiles **x** (above a specified threshold), this would support using the surrogate as a replacement for the primary outcome. In contrast, if the PTE varies across **x** and crosses the threshold, then the identified covariate profiles provide actionable guidance for the design of future studies that seek to leverage surrogates to reduce cost or participant burden – common motivations for surrogate evaluation [55], [56], [57]. For example, if the surrogate is strong for a particular covariate profile, a future study might measure and use the surrogate in place of the primary outcome for participants with that profile, while instead measuring the primary outcome for others. Treatment effects could then be assessed by combining information from both surrogate and primary outcome measurements. In this setting, a key design choice is the selection of the threshold *κ* used to define a sufficiently strong surrogate. A larger *κ* yields greater confidence in the surrogate’s validity but necessitates more extensive primary outcome measurement, whereas a smaller *κ* may offer greater cost savings at the risk of not capturing the treatment effect. To operationalize such cost savings at a chosen threshold *κ*, one could build on recent work by [58], who developed efficient testing procedures that integrate surrogate and primary outcome information from disjoint population subsets in randomized settings. Extending these ideas to observational studies, while appropriately accounting for confounding and selection bias, could enable more efficient study designs that strategically combine surrogate and primary outcome measurements across heterogeneous populations.

## Figures and Tables

**Figure A1:**
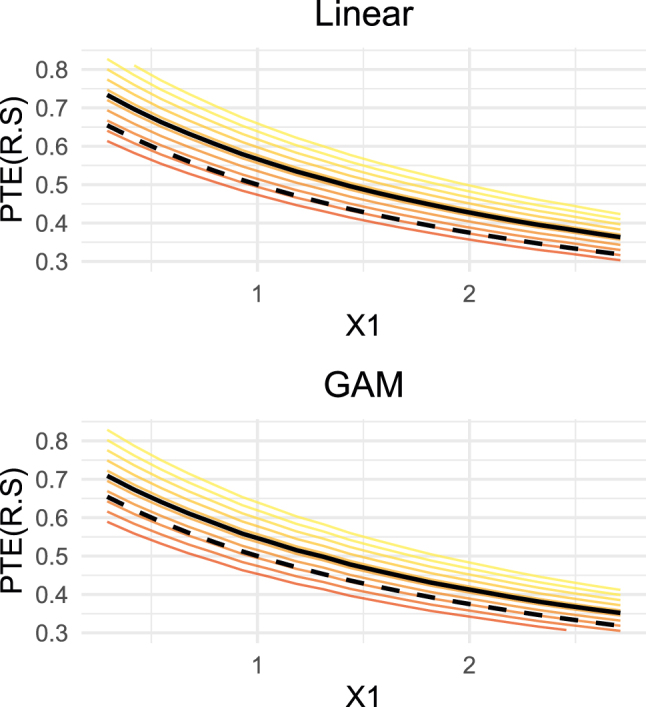
Sensitivity analysis results showing the estimated *R*_*S*_(**x**) plotted against *X*_1_ for Setting 1 with assumptions violated, displaying the biased estimate (black solid line), the truth (black dashed line), and bias-corrected values of $R_{S}^{\nu }\left(x\right)$ with *ν*_0_ = 0 and *ν*_1_ ranging from [−0.1, 0.1] times the standard deviation of *Y* (colored yellow to red where yellow indicates small values of *ν*_1_ and red indicates large values of *ν*_1_).

**Figure A2:**
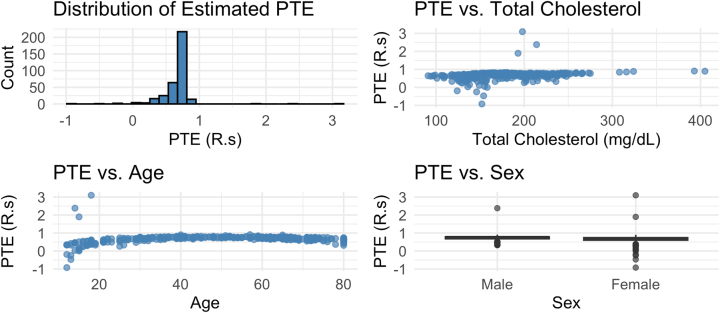
Estimation results for the NHANES survey data using GAMs as the base learners, evaluating the strength of HbA1c as a surrogate marker for plasma fasting glucose, when the exposure is obesity status; subfigures show the distribution of PTE estimates (top left panel) and PTE estimates by cholesterol (top right), age (bottom left), and sex (bottom right).

## Appendix A

In this section we propose a sensitivity analysis to examine violation of Assumption (C4) which is *Y* ^(1)^ ⊥ *S* ^(0)^|*S* ^(1)^, **X** and *Y* ^(0)^ ⊥ *S* ^(1)^|*S* ^(0)^, **X**. Recall that the quantities of interest were initially defined as:

$$\begin{array}{ll} \Delta \left(x\right) & =E\left(Y^{\left(1\right)}|X=x\right)-E\left(Y^{\left(0\right)}|X=x\right),\;\text{and}\\ \Delta_{S}\left(x\right) & =\int E\left(Y^{\left(1\right)}-Y^{\left(0\right)}|S^{\left(1\right)}=S^{\left(0\right)}=s,X=x\right)dF_{S^{\left(0\right)}|X}\left(s\right), \end{array}$$

and the PTE as *R*_*S*_(**x**) = 1 − Δ_*S*_(**x**)/Δ(**x**). We subsequently state in the main text that under our assumptions, we have

$$\begin{array}{ll} \Delta_{S}\left(x\right) & =\int E\left(Y|S=s,X=x,G=1\right)dF_{S|X,G=0}\left(s\right)\\ & \;-\int E\left(Y|S=s,X=x,G=0\right)dF_{S|X,G=0}\left(s\right); \end{array}$$

where in this section only we will use $\Delta_{S}^{A}\left(x\right)$ to refer to the expression above, and $R_{S}^{A}\left(x\right)$ to refer to the corresponding PTE, where “A” refers to Assumptions (because these are the identified quantities under our assumptions). That claim comes from the following:

$$\begin{array}{ll} \int E\left(Y^{\left(1\right)}|S^{\left(1\right)}=S^{\left(0\right)}=s,X=x\right)dF_{S^{\left(0\right)}|X}\left(s\right) & =\int E\left(Y^{\left(1\right)}|S^{\left(1\right)}=s,X=x\right)dF_{S^{\left(0\right)}|X}\left(s\right)\\ & =\int E\left(Y|S=s,X=x,G=1\right)dF_{S|X,G=0}\left(s\right) \end{array}$$

(and similarly for $\int E\left(Y^{\left(0\right)}|S^{\left(1\right)}=S^{\left(0\right)}=s,X=x\right)dF_{S^{\left(0\right)}|X}\left(s\right)$ ) where the first equality follows from (C4) and the second equality follows from (C3). There are many ways in which these assumptions could be violated, and thus these equalities would not hold. Following the spirit of existing work in this area [15], [16], [17], [18, 59], we propose to consider sensitivity parameters, defined as

$$\nu_{1}=\int E\left(Y^{\left(1\right)}|S^{\left(1\right)}=S^{\left(0\right)}=s,X=x\right)dF_{S^{\left(0\right)}|X}\left(s\right)-\int E\left(Y|S=s,X=x,G=1\right)dF_{S|X,G=0}\left(s\right)$$

$$\nu_{0}=\int E\left(Y^{\left(0\right)}|S^{\left(1\right)}=S^{\left(0\right)}=s,X=x\right)dF_{S^{\left(0\right)}|X}\left(s\right)-\int E\left(Y|S=s,X=x,G=0\right)dF_{S|X,G=0}\left(s\right)$$

such that if the assumptions hold, then *ν*_1_ = 0 and *ν*_0_ = 0. We do not consider *ν*_1_ and *ν*_0_ to be functions of *s* or **x** for simplicity. We can then identify the bias-corrected PTE for fixed values of *ν*_1_ and *ν*_0_ as

$$R_{S}^{\nu }\left(x\right)=1-\frac{\Delta_{S}^{\nu }\left(x\right)}{\Delta \left(x\right)}=R_{S}^{A}\left(x\right)-\frac{\left\{\nu_{1}-\nu_{0}\right\}}{\Delta \left(x\right)}$$

where $\Delta_{S}^{\nu }\left(x\right)=\Delta_{S}^{A}\left(x\right)+\left\{\nu_{1}-\nu_{0}\right\}$ such that {*ν*_1_ − *ν*_0_} reflects the bias in Δ_*S*_(**x**) and {*ν*_1_ − *ν*_0_}/Δ(**x**) reflects the bias in the PTE. One may consider examining resulting estimates across a range of values for *ν*_1_ and *ν*_0_ (for example, [−0.1, 0.1] times the standard deviation of *Y*) and/or determine which values of *ν*_1_ and *ν*_0_ would result in an estimated PTE below (or above) some threshold. We illustrate such an analysis using Setting 1 in our simulation study. We purposefully violated these assumptions by removing *X*_2_, *X*_3_, *X*_4_, and *X*_5_ from the estimation procedures. Figure A1 shows the results across 1,000 replications for the linear and GAM base learners. This figure displays the estimate (black solid line) which is biased, the truth (black dashed line), and calculated values of $R_{S}^{\nu }\left(x\right)$ fixing *ν*_0_ = 0 and *ν*_1_ ranging from [−0.1, 0.1] times the standard deviation of *Y* (colored yellow to red where yellow indicates small values of *ν*_1_ and red indicates large values of *ν*_1_), which extend across the truth. Such an approach may be useful to gauge the potential impact of violation of these assumptions. Future work in this area, specific to surrogates, is warranted and we hope to contribute to developing methods and tools for such sensitivity analyses in the future. In particular, expanding this type of sensitivity analysis to consider *ν*_1_ and *ν*_0_ as functions of *s* and **x**, as well as using the analyses to propose a potential testing framework for these assumptions, as is considered in Ding & Lu [15] would be especially useful.

## Appendix B

In this section, we examine simulation results in Settings 1–3 using a Monte Carlo (MC) approach to integration instead of our plug-in approximation, which relies on Condition (C6). Specifically, for each **x**, we generated *n* samples ${\left\{S_{j}^{\left(0\right)}\right\}}_{j=1}^{n}$ by adding resampled residuals to the predicted conditional mean from the surrogate model, i.e.,

$$S_{j}^{\left(0\right)}={\hat{\zeta }}_{0}\left(x\right)+\epsilon_{j}^{*},$$

where $\epsilon_{j}^{*}$ are sampled from the residuals of the control group. We then approximated the integral as

$$\frac{1}{n}\overset{n}{\underset{j=1}{\sum }}{\hat{\mu }}_{g}\left(S_{j}^{\left(0\right)},x\right),$$

where ${\hat{\mu }}_{g}\left(S_{j}^{\left(0\right)},x\right)$ denotes the predicted outcome under treatment *g*. This Monte Carlo approach explicitly evaluates the integral over the conditional distribution of *S* ^(0)^ and thus provides a direct comparison that accounts for both sources of approximation error: the error introduced by using a plug-in at the conditional mean rather than integrating over *S* ^(0)^, and the error inherent in estimating the full conditional distribution *F*_*S*∣**X**,*G* = 0_.

We compared these results to those obtained using the plug-in approximation based on Condition (C6), shown in Table A1, and found them to be very similar. These findings indicate that, for the learners examined and the settings considered, the plug-in approximation introduces minimal additional error relative to a fully Monte Carlo–based evaluation of the integral, while avoiding the substantial computational burden and statistical difficulty of explicitly estimating the conditional distribution.

**Table A1:** Simulation results for *R*_*S*_(**x**) in Setting 1–3, averaged over *X*_1_, comparing the plug-in approximation (proposed approach) versus Monte Carlo (MC) integration; Bias reflects the absolute value of the difference between the estimate and the truth, summarized as the median of 500 iterations; MAD reflects the empirical median absolute deviation (calculated as the median absolute deviation of estimates across the 1,000 simulation iterations, using the mad() function in R).

	Setting 1 (original)		Setting 1 (MC integration)	
	Linear	GAM	Linear	GAM
Bias	0.015	0.021	0.015	0.022
MAD	0.023	0.032	0.025	0.044
	**Setting 2 (original)**		**Setting 2 (MC integration)**	
	**Linear**	**GAM**	**Linear**	**GAM**

Bias	0.058	0.034	0.059	0.035
MAD	0.038	0.051	0.044	0.072
	**Setting 3 (original)**		**Setting 3 (MC integration)**	
	**Linear**	**GAM**	**Linear**	**GAM**

Bias	0.057	0.042	0.058	0.042
MAD	0.035	0.062	0.043	0.219

## Appendix C

Here, we describe in detail the data generating process for our simulation settings. In addition to Settings 1–3 described in the main text, here we include an additional Setting 4 that features *no* heterogeneity in terms of PTE, as a control setting.

In all settings, the baseline covariates are *X*_1_ ∼ *U*(0, 3), *X*_2_ ∼ Gamma(shape = 2, scale = 2), *X*_3_ ∼ *U*(0, 5), *X*_4_ ∼ Gamma(shape = 3, scale = 1), *X*_5_ ∼ *U*(0, 2), *X*_6_ ∼ Gamma(shape = 1, scale = 1). Treatment assignment is not randomized and instead depends on the baseline covariates. Specifically, we let *p*_*G*_ = *logit* ^−1^(0.2*X*_1_ + 0.3*X*_2_ + 0.5*X*_3_ + 0.2*X*_4_ + 0.4*X*_5_ + 0.1*X*_6_), and then *G* ∼ Bernoulli(0.5*p*_*G*_). Note that this construction allows treatment assignment to depend on **X**, but in such a way that we can easily tune the overall proportion assigned to treatment versus control, via the coefficient that is multiplied by *p*_*G*_. In our simulation settings, this resulted in roughly equally sized treatment and control groups.

In Setting 1, the surrogate is given by *S* = 1.5*G* + 0.2*X*_1_ + 0.2*X*_2_ + 0.3*X*_3_ + 0.1*X*_4_ + 0.4*X*_5_ + 0.3*X*_6_ + *N*(0, 0.4 + 1.4*G*). In Settings 2–4, the surrogate is given by *S* = 1*G* + 0.2*X*_1_ + 0.2*X*_2_ + 0.3*X*_3_ + 0.1*X*_4_ + 0.4*X*_5_ + 0.3*X*_6_ + *N*(0, 0.4 + 1.4*G*). In Setting 1, the linear model holds and thus, should perform well. Specifically, the primary outcome is given by *Y* = *G* + 2*S* + 0.2*X*_1_ + 0.5*X*_2_ + 0.2*X*_3_ + 0.1*X*_4_ + 0.3*X*_5_ + 0.4*X*_6_ + 2*GX*_1_ + *N*(0, 1). In Setting 2, the linear model is no longer correct, but the terms are additive and therefore the GAM should perform well. The primary outcome for Setting 2 is given by $Y=G+2S+\sin\left(X_{1}\right)+\cos\left(X_{2}\right)+X_{3}^{2}+X_{4}+\log\left(X_{5}+1\right)+\sqrt{X_{6}}+1.5GX_{1}^{2}+N\left(0,1\right)$ . In Setting 3, the terms are no longer additive and thus neither the linear nor the GAM assumptions hold. Specifically, the primary outcome for Setting 3 is given by $Y=G+2S+0.5X_{1}X_{5}^{2}+\log\left(X_{2}/X_{3}\right)+2\;\sin\left(X_{4}+X_{6}\right)+1.5GX_{1}^{2}+N\left(0,1\right)$ . The primary outcome *Y* in Setting 4 is the same as Setting 1, but without the interaction term for *G* and *X*_1_, so there is no heterogeneity in the PTE. That is, in Setting 4, *Y* = *G* + 2*S* + 0.2*X*_1_ + 0.5*X*_2_ + 0.2*X*_3_ + 0.1*X*_4_ + 0.3*X*_5_ + 0.4*X*_6_ + *N*(0, 1). The results from Setting 4 are included below in Table A2. Similar to the results in the main paper, we see reasonable performance in terms of coverage levels close to 95 % and small MSE.

**Table A2:** Simulation results for *R*_*S*_(**x**) in Setting 4, averaged over *X*_1_, where Bias reflects the absolute value of the difference between the estimate and the truth, summarized as the median of 500 iterations; MAD reflects the empirical median absolute deviation (calculated as the median absolute deviation of estimates across the 500 simulation iterations, using the mad() function in R); MAD^*b*^ reflects the median of the bootstrap estimates of MAD; MSE represents the median squared error; Coverage indicates the coverage rate of 95 % bootstrap confidence intervals with respect to the truth.

	Setting 4	
	Linear	GAM
Bias	0.025	0.036
MAD	0.038	0.053
MAD^*b*^	0.043	0.087
MSE	0.001	0.001
Coverage	0.969	0.990

## Appendix D

Our main example in Section 6 uses linear base learners to illustrate our proposed approach; here we examine using GAMs applied to the same NHANES data set, with results shown in Figure A2. The estimated PTE remained high, suggesting that the surrogate is strong for most patients. However, GAMs produced more outliers in the PTE estimates compared to the results using linear base learners. Unsurprisingly, these outliers correspond to the smallest estimates for Δ(**x**), which are close to (but not equal to) zero.

The suggested relationship between PTE and total cholesterol was similar across the two learners, with PTE increasing slightly as cholesterol increases. The relationship between PTE and sex was less pronounced in GAMs compared to linear models. The most notable difference appeared in the age covariate: while linear base learners showed higher PTE at younger ages, GAMs indicated higher PTE for middle-aged subjects with lower values for both young and old individuals.

Without knowing the ground truth, it is impossible to determine which base learner most faithfully represents reality in the example. Our simulations, however, provide guidance on the relative strengths and limitations of the learners. Linear models, while computationally efficient and interpretable, may fail to capture nonlinear relationships in certain covariate regions. The strong performance of the GAM learners in simulations suggests it can capture smooth nonlinear structure, such as nuanced age effects, that linear models miss. In applied work, the choice of learner should be informed not only by predictive performance but also by subject-matter knowledge about the likely form of treatment effect heterogeneity. Based on both the simulation evidence and the plausibility of smooth age effects in this application, we view the GAM results as the most reliable for this example, while recognizing that the best choice may differ in other contexts.
